# 1120. Absorption, Metabolism, and Excretion of [^14^C]-Tebipenem Pivoxil Hydrobromide (TBP-PI-HBr) Following a Single Oral Dose in Healthy Male Subjects

**DOI:** 10.1093/ofid/ofab466.1313

**Published:** 2021-12-04

**Authors:** Vipul K Gupta, Gary Maier, Leanne Gasink, Amanda Ek, Mary Fudeman, Praveen Srivastava, Angela Talley

**Affiliations:** 1 Spero Therapeutics, Inc., Cambridge, Massachusetts; 2 Maier Metrics and Associates, LLC, Worcester, Massachusetts

## Abstract

**Background:**

Tebipenem pivoxil hydrobromide (TBP-PI-HBr) is an oral prodrug that is converted to tebipenem (TBP), the active moiety, with activity against multidrug-resistant gram-negative pathogens, including extended-spectrum-β-lactamase (ESBL)-producing Enterobacterales. TBP-PI-HBr is the first oral carbapenem intended for treating complicated urinary tract infections and acute pyelonephritis. This study evaluated the absorption, metabolism, and excretion (AME) of TBP-PI-HBr following a single oral dose of [^14^C]-TBP-PI-HBr to healthy males and characterized metabolites in plasma, urine, and feces.

**Methods:**

This was a Phase 1, open-label, single-dose study in healthy subjects. Study drug was provided as radiolabeled and non-radiolabeled active pharmaceutical ingredient containing approximately 150 μCi of [14C]-TBP-PI-HBr. On Day 1, each subject received a 600 mg dose of TBP-PI-HBr. administered with 240 mL of water and fasted overnight for at least 10 hours. Blood samples were collected to determine TBP concentrations (whole blood), total radioactivity (whole blood and plasma), and metabolite profiling and identification were determined from plasma, urine, and feces. For mass balance, total radioactivity derived from urine and feces collections were determined. PK parameters were calculated using noncompartmental methods.

**Results:**

Total radioactivity in plasma and whole blood decreased rapidly with geometric mean t_½_ values of 6.0 hours and 3.5 hours, respectively and T_max_ of 1 hour. The cumulative mean recovery of radioactivity was 38.7% in urine and 44.6% in feces. Most of the administered radioactivity was recovered in the first 144 hours post dose in urine and feces (80.0%). Six of 8 subjects achieved a mass balance recovery ranging from 80.1% to 85.0%. The TBP plasma to total radioactivity ratio of 0.536 indicated that other metabolites contribute to the total radioactivity AUC in plasma. Metabolite profiling and identification results indicated that TBP was the major component in plasma and urine. The inactive ring open metabolite of TBP (LJC 11,562) was also found in plasma

( >10%), urine (5.27%), and feces ( >10%) as a secondary metabolite.

**Conclusion:**

This study adequately characterized the AME of TBP-PI-HBr in humans.

**Disclosures:**

**Vipul K. Gupta, Ph.D.**, **Spero Therapeutics** (Employee, Shareholder) **Gary Maier, PhD**, **Spero Therapeutics, Inc.** (Consultant) **Leanne Gasink, MD**, **Spero Therapeutics, Inc.** (Consultant) **Amanda Ek, MS**, **Spero Therapeutics, Inc.** (Employee) **Mary Fudeman, BA, MBA**, **Spero Therapeutics, Inc.** (Employee) **Praveen Srivastava, MS, BS**, **Spero Therapeutics, Inc.** (Employee) **Angela Talley, MD**, **Spero Therapeutics, Inc.** (Employee)

